# Does mindfulness change the mind? A novel *psychonectome* perspective based on Network Analysis

**DOI:** 10.1371/journal.pone.0219793

**Published:** 2019-07-18

**Authors:** Pablo Roca, Gustavo G. Diez, Nazareth Castellanos, Carmelo Vazquez

**Affiliations:** 1 Clinical Psychology Department, School of Psychology, Complutense University of Madrid, Madrid, Spain; 2 Nirakara Institute and Niraka Chair (Complutense University), Madrid, Spain; Universidad Rey Juan Carlos, SPAIN

## Abstract

If the brain is a complex network of functionally specialized areas, it might be expected that mental representations could also behave in a similar way. We propose the concept of ‘psychonectome’ to formalize the idea of psychological constructs forming a dynamic network of mutually dependent elements. As a proof-of-concept of the psychonectome, networks analysis (NA) was used to explore structural changes in the network of constructs resulting from a psychological intervention. NA was applied to explore the effects of an 8-week Mindfulness-Based Stress Reduction (MBSR) program in healthy participants (N = 182). Psychological functioning was measured by questionnaires assessing five key domains related to MBSR: mindfulness, compassion, psychological well-being, psychological distress and emotional-cognitive control. A total of 25 variables, covering the five constructs, were considered as nodes in the NA. Participants significantly improved in most of the psychological questionnaires. More interesting from a network perspective, there were also significant changes in the topological relationships among the elements. Expected influence and strength centrality indexes revealed that mindfulness and well-being measures were the most central nodes in the networks. The nodes with highest topological change after the MBSR were attentional control, compassion measures, depression and thought suppression. Also, cognitive appraisal, an adaptive emotion regulation strategy, was associated to rumination before the MBSR program but became related to mindfulness and well-being measures after the program. Community analysis revealed a strong topological association between mindfulness, compassion, and emotional regulation, which supports the key role of compassion in mindfulness training. These results highlight the importance of exploring psychological changes from a network perspective and support the conceptual advantage of considering the interconnectedness of psychological constructs in terms of a ‘psychonectome’ as it may reveal ways of functioning that cannot be analyzed through conventional analytic methods.

## Introduction

### Network theory and psychological functioning

Network theory (NT) has been used to describe the structure and functioning of dynamic complex systems by using Graph Theory [1,2]. The basic idea of NT is that systems can be represented as patterns of non-overlapping elements (represented as nodes) which are interconnected (represented as edges). The graph summarizes the pattern of relations among the elements in a network’s topology [3].

Current conceptualizations of brain functioning use NT to describe the complex functioning of neural circuitries and their connection to different type of data (e.g. performance in cognitive tasks) which has opened the field of ‘network neuroscience’ [4]. Within this framework, it is nowadays widely assumed that brain operates as a network and such organization underlies information processing, emotions, sensations, or thoughts [5–7]. However, in standard current network approaches, such mental representations are still considered as independent entities for which, at least for some aspects of them (e.g. fear reaction, reward and episodic memory), neuroscience research has found underlying networks of neural activity [8,9]. Thus, unfortunately, psychological constructs are still far from being perceived as a psychological network itself. For instance, whereas attentional tasks are linked to ventral- and dorsal-attention networks [10], the connection between attention, and other cognitive components (e.g. working memory), or emotional components (e.g. anxiety) is almost entirely unknown [10,11].

NT and network analyses have also been recently used as an innovative framework to understand psychopathology [12]. The literature of NT in psychopathology has grown very rapidly in the last few years and has being applied to explore a relatively large variety of psychological problems as shown in a recent systematic review [13]. From a network perspective, psychological disorders are not entities leading to symptoms (as it has been commonly assumed in traditional causal models of mental disorders). Rather, psychological disorders are understood as networks of elements (basically, symptoms and signs) which are pairwise associated forming a dynamically complex system with potential mutual casual influences. Psychological disorders, according to this point of view, could be nothing else that the very network of interconnected symptoms [14,15]. The radical departure of network theory from other current diagnostic approaches is that it defines mental disorders as conditions consisting of strongly connected symptom networks, with no assumption of a latent entity subsumed under the symptoms [14]. Nevertheless, some network theorists have recently suggested that dwelling excessively on the lack of interest of latent variables may misguide the focus from what is more promising in conceptualizing disorders from a NA perspective (i.e., the idea of causally interconnected elements) [16,17]. In this sense, the use of psychological constructs (e.g., traits assessed by questionnaires), and not only elements like symptoms or signs, is beginning to be used in NA (e.g., [18–20]) and it could also be possible that, in the future, hybrid models, using not only elements like signs and symptoms but also latent variables, could shed light on the connections between psychological elements [21,22].

NT provides several advantages over previous models of psychopathology. Firstly, network analyses allow to empirically identify which nodes have a central role within the network, which departs from the Diagnostic and Statistical Manual’s unproved assumption that all symptoms have the same diagnostic weight [23]. Secondly, and even more important, when longitudinal data are gathered, network analyses allow to explore potential causal links between the elements of the network [12,24], thereby identifying etiological pathways between symptoms (e.g. a sleep problem may lead to fatigue which leads to lack of energy and, finally, to anhedonia). Thirdly, network analyses also allow to explore time-related changes in the configuration of a given network which underlines the dynamic nature of psychological problems. These changes, that can be analyzed with mathematical tools [25], might be due to the mere passage of time or, more importantly, being the product of an intervention like the automatization of a cognitive task [26] or a psychological intervention [27,28]. Thus, analysis of different layers of networks along a temporal dimension (which may vary from milliseconds to months or years, depending on the type of study) allows to explore dynamic reorganizations of the elements of a network [25] which, in turn, may allow to identify key elements that can be targeted to promote changes in the network [29].

In sum, NT provides unique conceptual and analytic tools to understand in richer ways than in current prevailing models, the nature and etiology of mental disorders [15,30]. Expanding this approach to psychological models, in general, NT could provide a useful innovative paradigm to understand normal psychological functioning and its structure in a true holistic view [31].

### The proposal of a ‘*psychonectome’*

Most of the psychology, psychiatry, and clinical and cognitive neuroscience experimental studies are based on gathering information from questionnaires or tasks to measure skills, knowledge, abilities, attitudes or personality traits with the aim to identify cognitive or clinical deficits and to find all type of behavioral, neurobiological, or contextual correlates. The way in which typically operates this approach of doing science is by assuming that scores from these tests somehow quantify the status of a particular psychological construct [32–34]. For example, it is assumed that a test measuring memory skills taps a construct relatively independent than another test measuring attention. Yet, despite this implicit assumption, the nature of mental functions and mental representations is more likely to be one of interconnectedness where each component operates in relation to others having complex dynamics of mutual relations. These mental functions and representations do not exist as absolute constructs that operate as independent modules. As it has happened in the history of neuroscience in relation to the understanding of the nature of neural activity (for a review see [35]), localizationism should be abandoned in psychology in favor of considering mind as a complex network of psychological constructs.

The network perspective has been fruitfully used in fields as diverse as the understanding of brain structures and functioning, mental disorders, microbiota or social interactions, to name a few [36–38]. Yet, NT could also be expanded to grasp a more complex view on the mutual dependencies of elements of psychological functioning. In the psychological realm, expanding the recent attempts to understand psychological disorders using NT, it could be hypothesized that psychological functioning might be modeled as a complex network of psychological variables or modules that may [39], or may not [40], be interconnected. Furthermore, using appropriate designs and analyses, that network might reveal that some elements causally influence others.

A network of psychological elements, following what Guloksuz et al. [41] have called the wave of the ‘-omics’ approach (e.g. connectomics, genomics, or even ‘symptomics’ [42]), could be labelled as *psychonectome*. In general, a functional ‘connectome’ is the name given to those networks that reflect the activity of neural elements along a temporal dimension connecting neurons or regions (Friston, 2011; Korzeniewska et al., 2011). This psychonectome could be defined as a complex ensemble of dependences between psychological constructs (e.g. visuospatial memory, selective attention, or emotion regulation skills). That network might be, in turn, connected to more basic layers of elements belonging to the individual level (e.g. neural activity networks, signs and symptoms) or even elements external to the individuals (e.g. stressors, environmental circumstances) but still with the ability of activating some elements of other layers.

One of the most relevant features of the proposed *psychonectome* is that, as it happens in any network approach, the focus of interest is transferred from the individual variables or constructs (e.g. a behavioral response, socio-demographic information, or the score obtained from a psychological or clinical test) to the relation between them. The strength of a network is based on its decentralization and the synergy between its components is stronger than the sum of them. This new conceptualization of how systems work is being used not only to understand the functioning of complex systems but could also be fruitfully applied to understand the dynamic interconnections between psychological constructs as conceptualized in the *psychonectome* proposal.

### A *psychonectome* approach to a mindfulness-based intervention

A good case where the idea of psychonectome could be applied is the field of mindfulness- based interventions (MBI) as they tap a variety of psychological constructs. Mindfulness (MF) is defined as a state of consciousness with a present-orientated attention, on purpose, and non-judging [43]. Although mindfulness meditation has its origin over 2500 years ago in the ancient Buddhist traditions [44,45], the incorporation of several secular practices of MF in the western world is quite recent [46] and has generated great interest in the scientific community and the general public alike [47].

Despite the wide range of research on meditation benefits to date [48], relatively few studies have attempted to examine the action mechanisms through which meditation produces its effects (for reviews see [49–54]. Most of the theoretical models published to date emphasize the central role of attention regulation, which is thought to underpin emotional and cognitive flexibility [52], which in turn improves the emotion regulation processes [55], and the ability to maintain non-judging awareness of thoughts, feelings and experiences. Theoretical models also emphasize the importance of body consciousness and changes in self–perspective [51,56], as well as the role of self-compassion [50] in the promotion of changes due to MBI practices.

The most widely evaluated MBI is the pioneer Mindfulness-Based Stress Reduction (MBSR; [43,57]). MBSR is a treatment program originally developed within a hospital context for the management of stress caused by chronic pain, and subsequently applied in an extensive variety of problems like the reduction of comorbid symptoms in other health problems such as fibromyalgia [58], mood changes in patients with cancer [59] or multiple sclerosis [60].

Several meta-analysis and a growing body of robust empirical evidence from randomized controlled trials show that MBI is a promising treatment for a variety of mental health problems, including anxiety disorder [61], stress [62], depression symptoms [63] and depression relapses [64], substance abuse [65], and eating disorders [66], among others.

In addition to reducing psychological symptoms, mindfulness practice has been also shown to have positive effects on psychological well-being in healthy participants [67], quality of life improvements [62], empathy, compassion and prosocial behaviors [68] and cognitive functioning [49,51].

### The present study

Given the complex nature of mindfulness interventions and the well-studied cognitive, emotional, and psychopathological components that have been delineated in current theoretical models of mindfulness [51,52,69], it was considered that psychological changes associated to the practice of mindfulness would be an excellent proof of concept of the psychonectome idea.

As far as we know, NA has not been applied yet to explore the relationships among different psychological variables before and after a standardized MBSR intervention. Thus, the aim of this study was to examine the MBSR’s impact on the network dynamics between mindfulness, compassion, well-being, psychological distress and emotional and cognitive control constructs, and how these constructs are reorganized after the intervention. Although this is likely the first study on network analysis applied to the field of mindfulness, we tested several inter-related hypotheses based on the extant evidence on the effects and mechanisms of MBSR. All the hypotheses are framed under two theoretical assumptions: The first one is that network analysis may reveal complementary information about the relation between psychological measures that cannot be inferred by standard univariate statistics (i.e. comparing pre and post scores on selected measures). The second assumption is network analysis is able to find the dependences between constructs rather than the study of the constructs per se.

In sum, the following hypotheses were set up: 1) as supported by the literature review [48,50,61,62], the MBSR would yield significant changes, in an adaptive direction, in mindfulness, compassion, psychological distress, psychological well-being, and emotional and cognitive control variables; 2) after the MBSR program the networks of these constructs would become topologically reorganized (as measured by network paths and topological parameters) expecting a higher connectivity, clustering and efficiency; 3) based on theoretical models of MBSR functioning [52] it was also hypothesized that mindfulness, emotion regulation and well-being constructs would increase their centrality in the resulting psychonectome after the MBSR; and, 4) it was expected that these constructs would regroup after the MBSR in psychologically meaningful sub-networks as calculated by means of community analysis.

## Methods

### Participants

**Fig 1:**
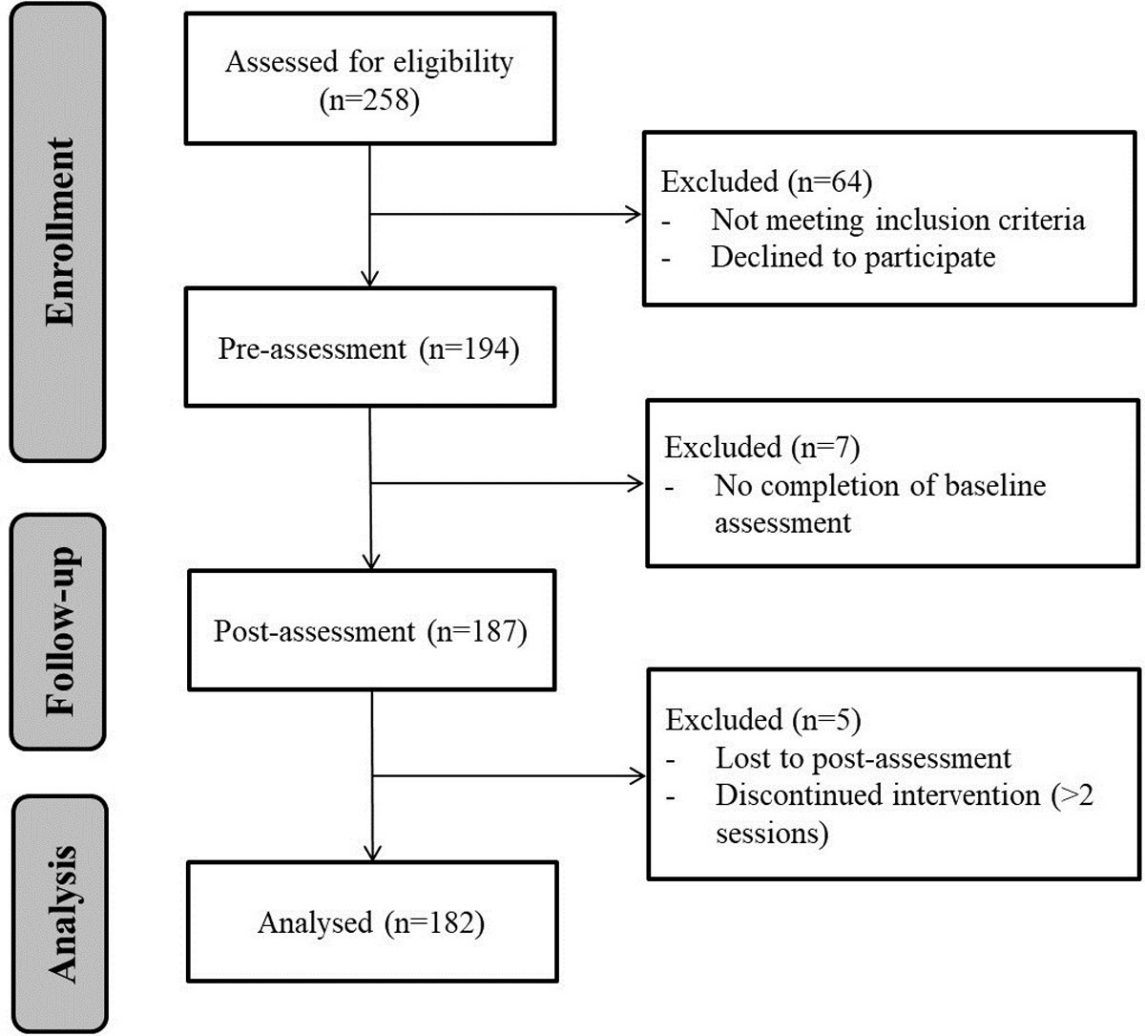
Participation flow diagram.

A sample of 258 adults, enrolled in a standardized 8-week MBSR program between April and December 2017, were invited to participate in this research (75.2% accepted to participate and fulfilled the inclusion criteria described below). After applying all exclusion criteria see Fig 1), data from a total of 182 individuals were included in all analyses. Participants mean age was 43.7 (*S*.*D*. = 9.77), 70.9% women, 95.7% had higher education, 40.1% married, 72.5% employed, 13,7% physical illness, 56% previous meditation experience and the meditation years average was 4.19 (*S*.*D*. = 5.72). The research study was approved by the university ethics committee prior to participant recruitment.

Inclusion criteria for MBSR program were as follows: 1) 18 years of age or more; 2) not having any current of serious psychological disorder or substance abuse / dependence. Statistical analyses were conducted only for data from participants who completed pre-assessment and attended a minimum of 6 sessions (i.e. 75% of the program). A precise description of the participation flow diagram is presented in Fig 1.

### Procedure and materials

The study followed a pre-post design where the participants were blind to the aims of the study. Participants were invited to participate at the moment they registered in the official website offering the MBSR course. Those who accepted to participate, were administered a brief online screening questionnaire on demographics and inclusion criteria and were asked to sign an informed consent. Then participants were asked to complete online (via Qualtrics software) the set of questionnaires described in detail in the next section. The online assessment was completed the week before starting of the program (baseline assessment) and during the week after the end of the MBSR (post-assessment). When necessary, reminders were scheduled for those participants who hadn’t completed the questionnaires. Each pre-post online evaluation lasted approximately 45 minutes. After completing the post-treatment assessments, participants were rewarded with an individualized report of their questionnaires scores.

#### Measures / Assessment

*Sociodemographic and health information*. For the baseline assessment a custom-made brief questionnaire, which included information about age, gender, education, occupation, psychological and physical health problems, was administered.

*Meditation experience*. A 32-item questionnaire, gathering information about previous meditation practice, meditation types and meditation retreats, was specifically designed for this study.

*Constructs associated to mindfulness training*. Five different areas, associated to the main outcomes and variables studied in the MF literature [52,70] were included. The constructs included:

- *Mindfulness* (i.e. facets and mindfulness, decentering, non-attachment, and bodily awareness);
- *Compassion* (i.e. compassion towards oneself and others and empathy)
- *Psychological well-being* (i.e. satisfaction with life, optimism, and overall well-being).
- *Psychological distress* (i.e. anxiety, stress, and depression)
- *Emotional and cognitive control* (i.e. emotional regulation, rumination, thought suppression and attentional control)

Table 1 shows a brief description of the measures and the internal consistency scores found in our study (Cronbach’s alpha based on the polychoric correlations).

**Table 1:** Constructs and instruments used in the study.

**Mindfulness**
- *Five-Facet Mindfulness Questionnaire-Short Form* (FFMQ, 20 items [α = .87]; [71]). It includes five component skills of of mindfulness: observing, describing, acting with awareness, non-judging of inner experience, and non-reactivity to inner experience - *Non-Attachment Scale* (NAS, 30 items [α = .93]; [72]). It measures the absence of fixation on thoughts, images, or sensory inputs, as well as an absence of internal pressure to get, hold, avoid, or change circumstances or experiences. - *Experiences Questionnaire* (EQ, 11 items [α = .89]; [73]). It assesses the ability to observe one’s thoughts and feelings in a detached manner. - *Multidimensional assessment of interoceptive awareness* (MAIA, 32 items [α = .94]; [74]). It measures interoceptive body awareness.
**Compassion**
- *Self-Compassion Scale-Short Form* (SCS-SF, 12 items [α = .88]; [75]). It measures three components of compassion to oneself: self-kindness, common humanity, and mindfulness. - *Compassion Scale* (CSP, 24 items [α = .86]; [76]). It assesses compassion to others, through the following components: kindness, indifference, common humanity, separation, mindfulness and disengagement. - *Interpersonal Reactivity Index* (IRI, 14 items [α = .77]; [77]). It measures empathy towards others. In this study only the Empathic Concern subscale was included.
**Psychological well-being**
- *Satisfaction With Life Scale* (SWLS, 5 items [α = .87]; [78]). This is a measure of global life satisfaction. - *Life Orientation Test–Revised* (LOT-R, 10 items [α = .67]; [79]). It measures dispositional optimism. - *Pemberton Happiness Index* (PHI, 11 items [α = .91]; [80]). A measure that includes both hedonic and eudaimonic components of psychological well-being.
**Psychological Distress**
- *Depression Anxiety Stress Scales* (DASS-21, 21 items [α = .92]; [81]). It measures symptoms of depression, anxiety and stress.
**Emotional and cognitive control**
- *White Bear Supression Inventory* (WBSI, 10 items [α = .89]; [82]). It measures unwanted intrusive thoughts and thought suppression. - *Ruminative Response Style* (RRS, 22 items [α = .92]; [83]). It assesses an excessive focus on causes and consequences of depressive symptoms. It includes two factors: reflection and brooding. - *Emotion Regulation Questionnaire* (ERQ, 10 items [α = .76]; [84]). It measures two emotional regulation strategies: reappraisal and suppression. - *Attentional Control Scale* (ACS, 20 items [α = .84]; [85]). It assesses perceived ability in executive control over attention.

### Mindfulness-based stress reduction program (MBSR)

The MBSR programs was implemented at a university-associated center specialized in MBI. MBSR instructors were highly experienced and all were certified by the University of Massachusetts Center for Mindfulness (https://www.umassmed.edu/cfm/). Each instructor had more than 6 years of teaching experience and had conducted a minimum of 30 MBSR programs.

The MBSR program [86] consisted of 32-hour training during eight weeks, including a 3-hour initial orientation session, 7 weekly 2.5-hour of face-to-face sessions, an 8-hour intensive day of practice, 45 minutes of daily home formal and informal practices and a final 3.5-hour session. Training was conducted in groups of 20–30 participants. During the program, different mindfulness practices are performed, including focused attention on the breath, open monitoring of awareness in body-scanning, prosocial meditation (i.e. loving kindness and compassion) and gentle hatha yoga. To support the practice, each participant was given a set of pre-recorded audio files to guide daily practices and a MBSR Workbook. Program adherence was supported through regular group supervision meetings.

## Data analysis

### Data preprocessing and univariate statistical test

Data pre-processing, analyses of missing data and imputation methods were conducted with the SPSS v. 22. All the network analyses were carried out with R v. 3.3.1 and Matlab R2017b.

Following CONSORT guidelines [87], Intent-To-Treat (ITT) analyses were carried out following Newman's guidelines [88]. Maximum Likelihood (ML) estimation was performed via Expectation Maximization imputation (EM) using SPSS v.22 software. We followed the procedure proposed by Hair and colleagues [89] to treat missing data. We first tested for both the recommended limits of missing measures [90], with a 10.8% of overall missing values and their random patterns using a Little MCAR test (χ2 (1674) = 356.45, p > .05), concluding that missing data were completely random. To examine whether missing data could be predicted by other variables (attrition bias) a logistic regression was carried out by including clinical (DASS-21 stress, anxiety and depression symptoms), demographic (gender, age, education), and meditation experience (meditation practice and years of experience). Only the stress score at baseline was weakly associated with greater likelihood of post missing data (*R*^2^ de Cox-Snell = 0.07; *R*^2^ de Nagelkerke = 0.10). However, the correlation between stress and likelihood of missing data wasn’t significant. None of the included variables predicted missingness. Finally, Maximum Likelihood estimation (ML) was performed, and Sensitivity Analysis compared the results of the completers to the estimated values was carried out, concluding that ML estimation would not lead to biased estimations.

All measures were statistically compared (pre versus post) using a Student *t*-test with SPSS as showed in Table 2.

**Table 2:** Paired comparisons of pre-post measures in the constructs assessed in the MBSR program. (Description of the variables and their acronyms is shown in Table 1).

	Pre		Post		
Node/Construct	Mean	SD	Mean	SD	t (181)
**Mindfulness**					
FFMQ-Observing	3.42	0.80	3.89	0.70	-10.19**
FFMQ-Describing	3.46	0.75	3.73	0.66	-6.40**
FFMQ-Acting Awareness	2.82	0.74	3.31	0.69	-10.47**
FFMQ-Non Judgment	3.40	0.90	3.97	0.69	-10.04**
FFMQ-Non Reactivity	2.96	0.60	3.49	0.62	-11.38**
NAS	4.25	0.76	4.68	0.71	-8.84**
EQ	3.23	0.58	3.79	0.59	-13.63**
MAIA	2.76	0.75	3.47	0.64	-16.91**
**Compassion**					
SCS- Self Kindness	5.96	1.82	7.38	1.57	-11.71**
SCS- Common Humanity	6.14	1.60	7.45	1.42	-11.42**
SCS- Mindfulness	5.94	1.60	7.59	1.45	-15.29**
CSP	4.26	0.45	4.35	0.42	-4.22**
IRI-Empathic Concern	28.24	4.13	28.35	3.91	-0.49
**Psychological well-being**					
SWLS	22.30	6.34	24.03	5.92	-6.67**
LOT	21.75	4.21	23.00	3.81	-5.85**
PHI	77.18	17.00	84.70	16.32	-8.51**
**Psychological Distress**					
DASS-Depression	0.60	0.59	0.32	0.35	7.36**
DASS-Anxiety	0.51	0.50	0.38	0.34	4.29**
DASS-Stress	1.15	0.59	0.76	0.46	9.50**
**Emotional and Cognitive Control**					
WBSI	32.54	8.10	28.79	7.94	7.56**
RRS-Brooding	9.80	3.16	8.39	2.37	7.72**
RRS-Reflection	10.88	3.03	10.32	3.03	3.13**
ERQ-Reappraisal	27.90	6.92	28.76	6.49	-1.83
ERQ-Suppression	11.20	5.17	10.08	4.52	4.22**
ACS	2.87	0.40	3.00	0.37	-5.59**

** = p < .001; SD = Standard deviation.

### Network analysis

NA was conducted to analyse the multiple relations (edges) between different psychological constructs (nodes) simultaneously, and how those relations would be reorganized after the MBSR. The successive steps procedure proposed for network analysis in psychology [91] was followed. Analyses were conducted adapting this procedure to our objectives: 1) pre and post MBSR network estimation; 2) pre and post MBSR network inference (topological characterization); and 3) pre and post MBSR network node communities analysis.

#### a) MBSR network estimation

The MBSR networks structure were estimated by using Gaussian Graphical Model (GGM) [92], a Regularized Partial Correlation Network (RPCN). Although the use of partial correlation in psychology has been recently questioned [93], this type of correlation has been typically used as a way to reduce spurious associations between variables. Due to the ordinal nature of the variables, a Spearman correlation matrix was used as input for the GGM (for a recent tutorial see: [94]). A network structure was estimated with the 25 nodes representing constructs associated to mindfulness training (Table 2) which resulted in 300 potential no symmetric connections (edges) among these nodes [([k*k−1]/2, being k the number of nodes], which can be either positive or negative depending on the direction of the correlation.

A RPCN has two important features. First, each edge represents partial correlations between nodes (conditional dependence relations). Thus, an association between two nodes indicates that they remain conditionally dependent after controlling for all other associations among the rest nodes in the network. If no edges emerge between two nodes, which means that the nodes are conditionally independent after controlling for the associations among all other nodes. Second, this procedure included the network regularization [95], a statistical strategy that uses a least absolute shrinkage (LASSO) correction to shrink connections in the network and sets small connections to zero. LASSO allows to reduce the number of false positive correlations within networks, which avoids spurious connections between nodes and facilitates the interpretation of the network structure [96]. The *Parcor* R-package [97] was used to implement the adaptive LASSO approach. The results were visualized using the *q-graph* R-package [98] and the *Fruchterman-Reingold algorithm* [99] to draw close those nodes with stronger and/or more connections and in the periphery those with low centrality. In order to quantify visually–based inferences of network architecture, we used the find-path algorithm (implemented in Matlab toolbox). In this algorithm, paths are defined as a sequence of linked nodes that never visit a single node more than once. Also, considering that our proposed ‘psychonectome’ included five general psychological constructs, it was thought that a network visualization using Principal Components Analysis, could be appropriate as an alternative parsimonious visualization method (see S1 Fig).

#### b) MBSR network inference (topological characterization)

In this second step, we computed different centrality parameters for the pre and post MBSR networks, as well as predictability analysis. Also, centrality plots were calculated, displaying the centrality of each node in the network. All centrality measures represent the connectedness of a given node with all other nodes in the network, assuming that highly connected nodes are usually more relevant in the network. There are some controversies on the adequacy of classical centrality indexes. For instance, strength, which is a commonly used centrality measure, is the sum of the absolute weights connected to each node, meaning that nodes with high strength are strongly or highly associated with other nodes in the network. Yet, these indexes are calculated based on the absolute values of edge-weights, which may distort the conclusions on the network structure, if there are negative relationships between nodes [100]. In our MBSR networks, mindfulness, compassion and well-being are expected to have negative relationships with psychological distress and most of psychological functioning nodes. To address this limitation, instead of strength we have included the node’s Expected Influence index (EI) [100], using *expectedinf* function from the R-package *networktools* [101]. EI is a new measure of node importance, being the sum of both positive and negative weights between a node and all other nodes in the network. Also, given the lack of reliability of other centrality indexes (e.g., betweenness and closeness) [102,103] we included other centrality measures: *a) Degree*: the number of connections per node. According to the degree, hubs are defined as those nodes with the highest degree; *b) Clustering*: it measures how close a node is to the other network nodes, sharing a clustering. High clustering means that a node’s neighbors are neighbors between them; c) *Efficiency*: The global efficiency is the average of inverse shortest path length, and is inversely related to the path length.

Predictability of nodes in the network was estimated using the R package *mgm* [104]. Predictability is an additional network measure, defined as the degree to which a given node can be “predicted” by all other nodes in the network. Whereas centrality parameters provide relative measures of interconnectedness, predictability can be considered an absolute measure of interconnectedness (i.e., how much variance of a node can be explained by other nodes in the network)–(S1 and S3 Figs in Supplementary materials). Finally, networks accuracy (i.e. resistance to sampling variation) and stability (i.e. whether the network interpretation remains stable with less observation) were calculated using R-package *bootnet* [94]–see S4 and S5 Figs in Supplementary materials.

#### c) MBSR network node communities

Finally, one way to analyze the reorganization of constructs (Table 1) can be done by means of the *community analysis* of the graph. A community is defined as a set of nodes that cluster more strongly amongst each other than with other nodes of the network. The network perspective explains such communities as a result of increased mutual influences among nodes in a given cluster. The community structure was explored using the Spinglass algorithm [105], with the R package *igraph* [106]. The following parameters were used: γ = 1, start temperature = 1, stop temperature = .01, cooling factor = .99, spins = 25.

## Results

### Pre-post psychological changes (Hypothesis 1)

To analyze the effects of the MBSR program on the constructs selected, a standard analysis of pre-post mean differences was performed. Table 2 lists the means score and standard deviation for each variable as well as the significant differences found. As it was hypothesized, there was a significant change in most of the dependent variables measuring mindfulness, compassion towards others and oneself, psychological well-being, and emotional and cognitive functioning, as well as a significant reduction of psychological distress. Thus, the MBSR program was effective in changing the psychological state of participating individuals as confirmed by standard statistical procedures, which is a common result in MBSR programs. The rest of the analyses conducted and reported in the next sections were aimed to go beyond this standard analytic strategy by using network analysis procedures.

### MBSR network estimation (Hypothesis 2)

**Fig 2:**
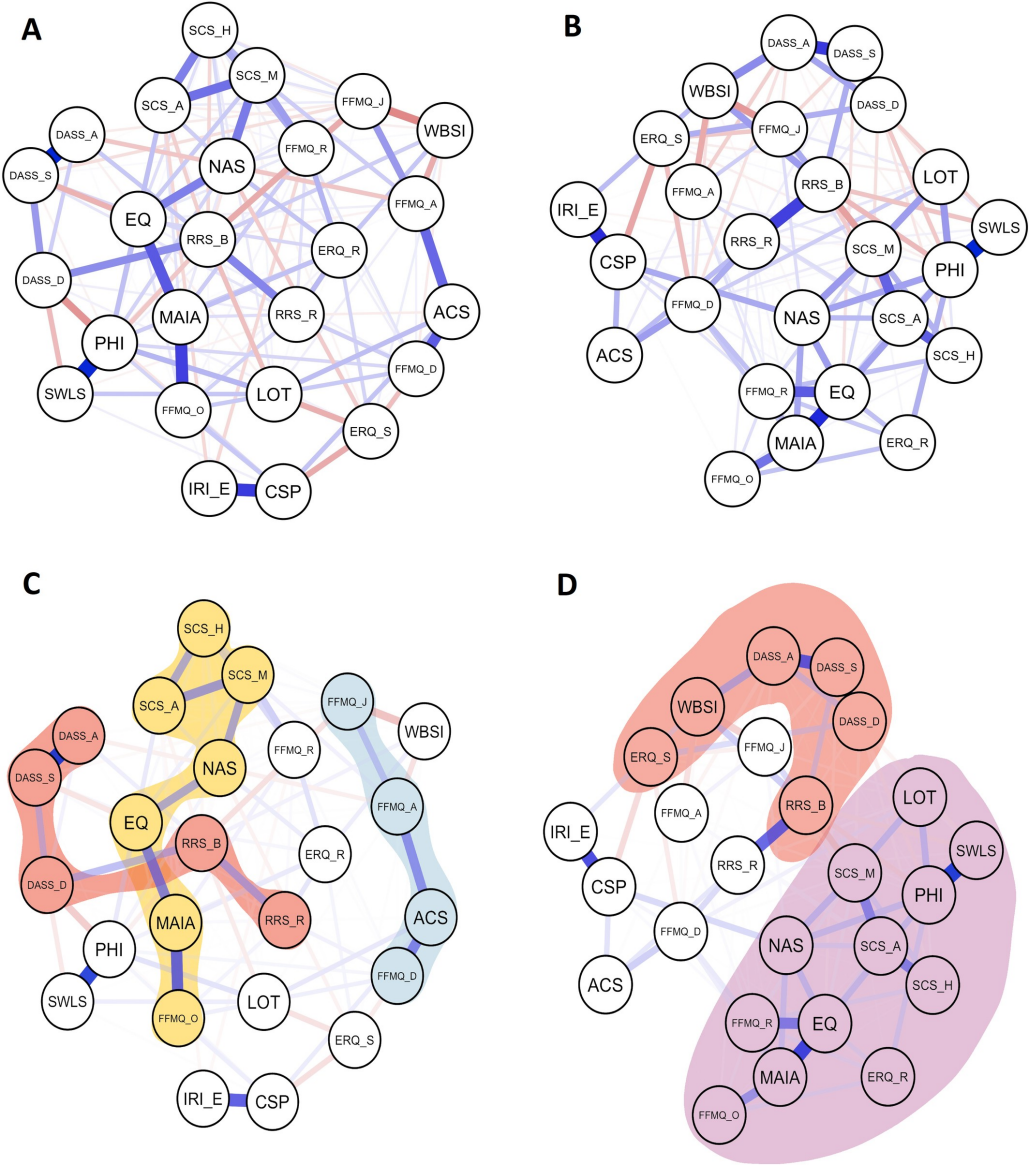
Network representations. Network representations in pre (left panels A and C) and post (right panel B and panel D) MBSR intervention. A network is graphed by nodes (circles representing the constructs assessed in the MBSR program, described in Table 1 and named in Table 2) and edges (lines representing the statistical correlation between nodes as described in Methods section). Blue edges represent positive relationships and red edges represent negative relationships. The spatial position of nodes is chosen by the Fruchterman-Reingold algorithm to draw close those nodes with stronger and/or more connections while it places in the periphery nodes with low centrality. A) Regularized partial correlation networks pre-MBSR and B) post-MBSR intervention. The figure also shows paths, defined as a sequence of linked nodes that never visit a single node more than once, in pre-MBSR (panel C) and post-MBSR (panel D).

Before inferring the network reorganization after the MBSR, the network architecture in both pre- and post-intervention was analyzed. The pre and post MBSR regularized partial correlation networks are presented in Fig 2. Of all the possible 300 edges, 43.67% and 40.67% were estimated to be different from zero in pre- and post-assessments, respectively, which means that neither an all-to-all network nor a disconnected topology was found.

A path is defined by the sequence of links between constructs and the corresponding strength of the dependence (shown here in parenthesis). Using a find path algorithm, three paths were identified in the pre-MBSR network (displayed in different colors in Fig 2, panel C and panel D):

1. A first path was found crossing transversely the network (shaded in yellow in Fig 2, Panel C). This path was formed by mindfulness and self-compassion measures: FFMQ-O—MAIA (0.33), MAIA—EQ (0.29), EQ—NAS (0.22), NAS—SCS-M (0.23), SCS-M—SCS-A (0.25), SCS-A—SCS-H (0.22).
2. A second path included clinical symptoms and rumination measures (shaded in red in Fig 2, Panel C): DASS-A–DASS-S (0.45), DASS-S–DASS-D (0.18), DASS-D–RRS-B (0.20), and RRS-B–RRS-R (0.21).
3. A third path included the rest of mindfulness measures and the self-reported attentional control measure (shaded in blue in Fig 2, Panel C): FFMQ-D–ACS (0.28), ACS–FFMQ-A (0.27), FFMQ-A–FFMQ-J (0.18).

Interestingly, some strongly related dyads (i.e. pairs of highly related constructs that are less dependent on the rest of the network) were also found. One dyad was related to self-compassion measures (IRI-E–CSP (0.34), whereas the rest were composed by measures related to distress and/or well-being: PHI–SWLS (0.42), DASS-S–DASS-A (0.45), WBSI—FFMQ-J (-0.20), and PHI—DASS-D (-0.19).

In regard to the network configuration after the MBSR, the three paths observed in the pre-MBSR network disappeared. Instead, the post-MBSR network appears reorganized in a different manner, emerging two visually differentiated sub-networks (see Fig 2) which were further mathematically explored (see the following Results section). A close inspection of the Fig reveals that in the upper half part of this post-MBSR network (shaded in red in Fig 2, Panel D), it appeared a subnetworks of nodes that are mostly related to psychopathological constructs [i.e. stress (DASS-S), anxiety (DASS-A), depression (DASS-D); thought suppression (WBSI); emotion suppression (ERQ-S); and rumination-brooding (RRS-B)]. In the lower area of the network (shaded in purple in Fig 2, Panel D), a second subnetwork emerged in which most of the nodes are adaptive psychological constructs (i.e. mindfulness, attentional control, self-compassion, compassion and psychological well-being). Thus, the overall reorganization of the networks indicates that the nodes of the different sets of measures were rather scattered or disconnected at pre-MBSR whereas, at post-MBSR, the nodes seem to be more closely reorganized.

Other six network features are noteworthy: 1) a psychologically interesting feature was that the “reappraisal” component of “Emotional Regulation Questionnaire” (ERQ-R; an adaptive emotional regulation strategy) was related to rumination (RRS-R), thought suppression (WBSI) and non-reactivity (FFMQ-R) at pre-MBSR; however, after the MBSR, reappraisal was related to mindfulness (EQ, FFMQ-R and FFMQ-O) and well-being measures (PHI); 2) in regard to the “suppression” component of “Emotional Regulation Questionnaire” (ERQ-S; a non-adaptive emotional regulation strategy–see [107]), which was with thought suppression (WBSI), depression (DASS-D) and Empathic Concern (IRI-E) after the MBSR, and maintained maintaining their negative relationships with compassion to others (CSP) and describing (FFMQ-D); 3) something similar happened with the “reflection” component of Ruminative Responses Scale (RRS-R; a short and mid-term adaptive strategy), which established positive relations with the describing facet of mindfulness (FFMQ-D) and attention control (ACS) after the MBSR; 4) another interesting feature was that the self-compassion measures (SCS) were quite disconnected from the well-being measures (PHI, SWLS and LOT) before the intervention but established strong connections after the MBSR; 5) in the same way, optimism (LOT), a variable that was quite disconnected from the rest of well-being measures (general well-being [PHI] and life satisfaction [SWLS]) before the intervention, increased its relations with them and with self-compassion after the MBSR; and 6) in post-MBSR network the thought suppression node (WBSI) established strong positive relations with other non-adaptive or psychopathological elements, such as emotional suppression (ERQ-S), brooding (RRS-B) and anxiety (DASS-A).

### MBSR network inference (topological characterization) (Hypothesis 3)

**Fig 3:**
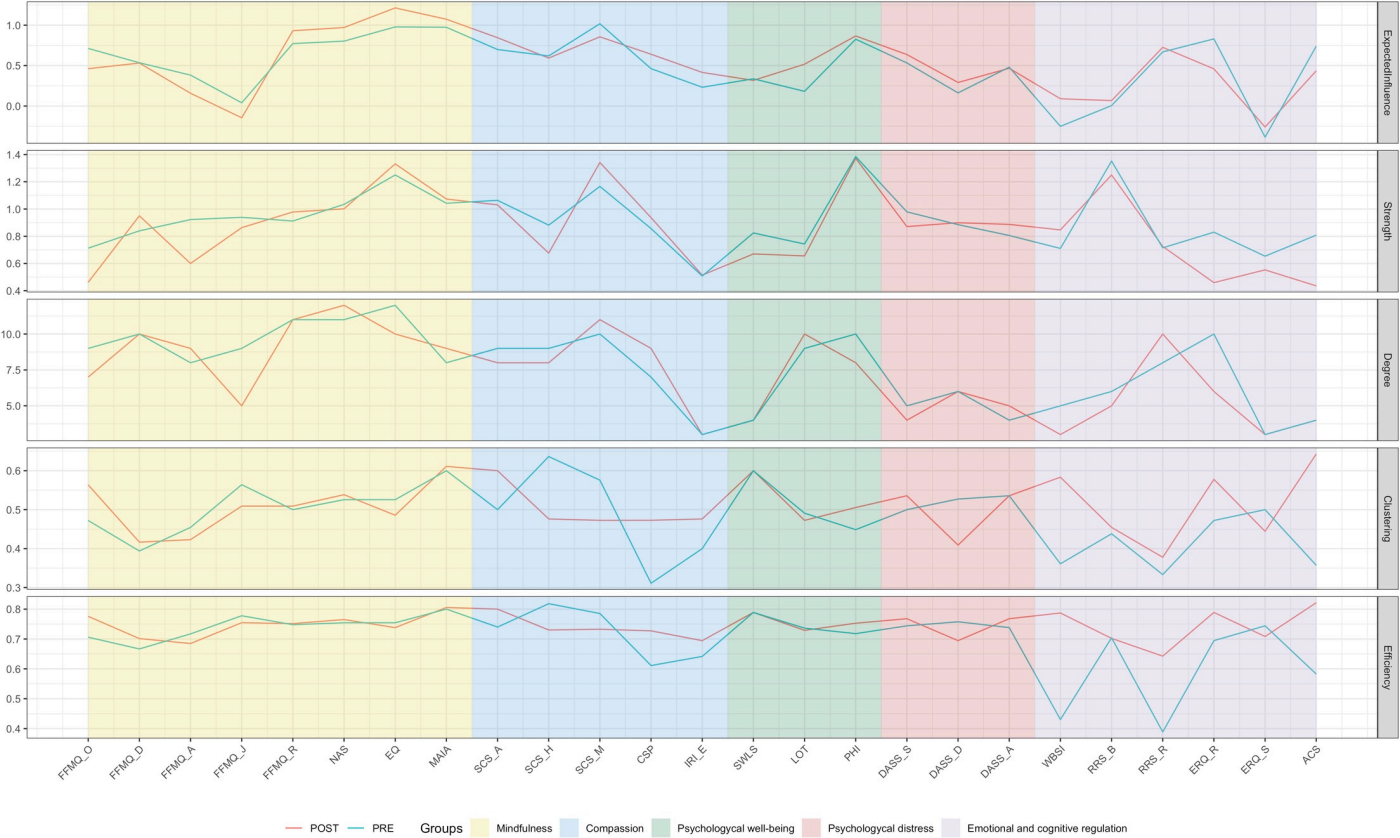
Topological characterization of networks. Topological characterization of pre- and post-MBSR intervention networks for Expected Influence and Strength. Constructs theoretically associated with MBSR are grouped according to classification given in Table 1. Each measure estimates the topological role of each node (x-axis, described in Table 2) in the network. A) Expected Influence: the sum of both positive and negative weights between a node and all other nodes in the network (nodes with high EI are positively strongly associated with other nodes in the network); B) Strength: sum of the absolute weights of all edges in the network involving that node (nodes with high strength are strongly associated with other nodes in the network); C) Degree: number of connections per node (the higher the degree, the more connected the network is); D) Clustering: closeness of a node to the other network nodes, sharing a clustering (high clustering means that a node’s neighbors are neighbors between them); E) Efficiency: average of inverse shortest path length (which is inversely related to the path length).

The Expected Influence (EI) and strength of all psychological constructs were calculated (see Fig 3). The constructs with the highest EI scores, both at pre- and post-MBSR were: mindfulness measures such as non-react (FFMQ_R), interoceptive awareness (MAIA), nonattachment (NAS), decentering (EQ), self-compassion mindfulness (SCS_M) and the overall well-being measure (PHI). Only in four nodes [brooding (RRS_B), reflection (RRS_R), non-judgment (FFMQ_J) and emotional regulation-reappraisal (ERQ_R)], there was a discrepancy between EI and strength indicating that in those nodes there were many negative edges. No major changes were observed after the MBSR in either EI or strength.

In addition to EI, other centrality measures were calculated (see Fig 3): *a) Degree*: the analysis of hubs, defined as sets of nodes with the highest degree, indicated a pattern highly consistent with the results on EI and strength, with the exception of optimism (LOT), which showed a high number of relations (i.e. degree), but with low weights (i.e. low EI and strength); b) *Clustering*: nodes with the highest topological change were common humanity (SCS_H), compassion to others (CSP), depression (DASS_D), thought suppression (WBSI), and attentional control (ACS); *c) Efficiency*: nodes with the highest changes in efficiency were thought suppression (WBSI), attentional control (ACS), and reflection (RRS_R). It must be noted that nodes with highest changes in either clustering or efficiency are interpreted as having a key role in the network reorganization.

Pre-post MBSR predictability values for each node are presented in Supplementary materials (S2 and S3 Figs). The average predictability in both networks was similar, rating from 0.55 (pre-MBSR) to 0.57 (post-MBSR), indicating that an overall average of 56% of the variance of a node was predicted by its neighbors at both moments of assessment. As compared to other network studies in psychology, following the criteria suggested by Haslbeck & Fried [104], the overall predictability was high. Also, additional analyses on the pattern of correlations between pre- and post-MBSR interventions on strength and degree, clustering and efficiency (see Supplementary Materials, Network inference) seemed to indicate that that there was a genuine network reorganization of psychological constructs after MBSR intervention.

### MBSR network node communities (Hypothesis 4)

**Fig 4:**
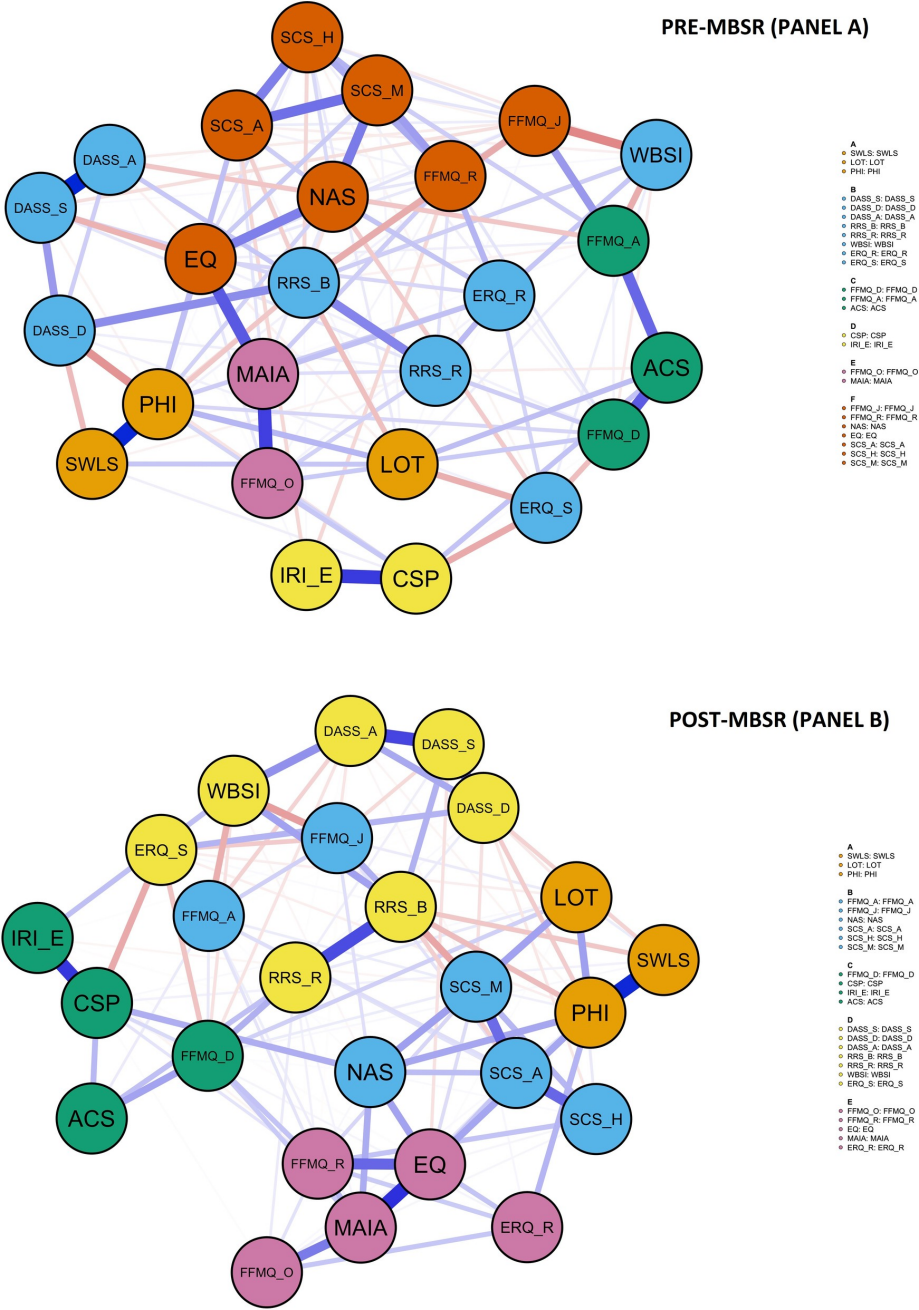
Community detection analysis. Results of the community detection analysis performed on the network shown in Fig 1. The identified communities depict the variables that more strongly inter-correlated.

Fig 4 shows the results of the Community Detection analysis. The Spinglass algorithm detected six node communities in pre-MBSR network and five node communities in post-MBSR network.

Six meaningful communities or clusters emerged in pre-MBSR network:

1. The largest cluster (Cluster B in blue in Fig 4), included 8 nodes that reflected psychological distress (anxiety [DASS-A], stress [DASS-S], and depression [DASS-D]) and emotional and cognitive control measures (brooding [RRS-B], reflection [RRS-R], reappraisal [ERQ-R], emotion suppression [ERQ-S] and thought suppression [WBSI]).
2. The second largest cluster F, depicted in red, included 7 nodes which all reflected mindfulness (decentering [EQ], non-attachment [NAS], non-reactivity [FFMQ-R], non-judgment [FFMQ-J] and mindfulness [SCS-M]) and self-compassion measures (common humanity [SCS-H] and self-kindness [SCS-A]).
3. The third largest cluster A (depicted in orange) included 3 nodes which integrates the well-being measures (general well-being [PHI], life satisfaction [SWLS] and optimism [LOT]).
4. The fourth largest cluster C (depicted in green) also include 3 nodes with other mindfulness measures (acting awareness [FFMQ-A], describing [FFMQ-D] and attentional control [ACS]).
5. Finally, two small clusters were found:
  - Cluster E (depicted in purple) composed by the rest of mindfulness measures (interceptive awareness [MAIA] and observing [FFMQ-O]).
  - Cluster D (depicted in yellow) composed by compassion measures (compassion to others [CSP] and empathic concern [IRI-E]).

These six large clusters were reorganized at post-MBSR, generating a 5-cluster final organization:

1. The mindfulness and self-compassion measures that were scattered in clusters F, C and E in pre-MBSR network, were reorganized in two big clusters after the MBSR:
  - Cluster B (in blue) included non-attachment [NAS], self-compassion [SCS], non-judgment [FFMQ-J] and acting awareness [FFMQ-A].
  - Cluster E (depicted in purple) included decentering [EQ], interoceptive awareness [MAIA], non-reactivity [FFMQ-R], observing [FFMQ-O] and reappraisal [ERQ-R].
2. The Psychological distress and Emotional and cognitive control measures remained grouped in the same cluster D (depicted in yellow), with the exception of emotional reappraisal [ERQ-R].
3. Well-being measures remained all together in orange cluster A (general well-being [PHI], life satisfaction [SWLS] and optimism [LOT]).
4. Finally, a mixed cluster C (depicted in green) was found, including compassion to others measures [CSP and IRI-E], attentional control [ACS] and describing [FFMQ-D].

## Discussion and conclusions

The current study belongs to the growing body of scientific literature using NA to explore alternative theoretical and empirical conceptualizations in psychological science [15,108]. Based on that background, we propose the ‘*psychonectome’* concept, defined as a complex ensemble of dependences between psychological constructs, where the focus of interest is transferred from the changes in individual constructs to the relation between them. The underlying principle for this proposal is that the functioning of the mind is viewed as a complex network of interrelated psychological constructs. As far as we know, this is the first empirical study using NA to explore the effects of a standardized mindfulness intervention (MBSR) on the reorganization of psychological constructs that are central to that practice.

Based on the extant evidence on the effects of MBSR programs [48–50], the first hypothesis of the study stated that the MBSR would yield significant adaptive changes by increasing mindfulness, compassion, well-being, emotion and attention regulation scores and reducing psychological distress and rumination. Using standard univariate statistics, the results consistently confirmed the first hypothesis. The MBSR yielded significant change in almost all the dependent variables selected in the expected direction. Yet, this analytic approach does not facilitate to inquire about the action mechanisms involved in the psychological changes produced by the practice of MBSR, which is something necessary to keep moving forward in this area [109].

To overcome those analytic limitations, the second hypothesis of the study, based on a network analysis approach, was that the MBSR would reorganize the network topology after the program. The results identified three main paths in the pre-MBSR network (i.e. mindfulness and self-compassion; clinical symptoms and rumination; and most of FFMQ mindfulness components with attentional control measure). Yet, these three paths disappeared at post-MBSR network, and instead a new reorganization appeared with two distinct visually differentiated sub-networks (see Fig 1). In the upper half area of this post-MBSR network, most of the nodes were related to psychopathological constructs while in the lower area of the network, most of the nodes were related to adaptive psychological constructs. Taking into account that the visual interpretation of networks must be cautious [110], we further mathematically explored this network reorganization by means of community analysis (hypothesis four).

Specifically, three network reorganization features are especially noteworthy: First, although compassion (both self and others) is an attitudinal foundation of mindfulness practice [86], and self-compassion is a significant predictor of therapeutic change in MBIs [111], these components are implicitly, but not explicitly, taught in MBSR practices [112] and it is questioned whether or not they should be an ingredient of these practices [113]. Our results showed that, while self-compassion elements were relatively disconnected from well-being measures before the intervention, they became strongly connected after the MBSR. Furthermore, community analysis detected that, after the MBSR, self-compassion measures behave similarly to some mindfulness constructs, such as non-attachment and non-judgment. These NA-based results suggest that self-compassion is indeed an important variable and may deserve to have a more active role in mindfulness programs.

A second interesting aspect of the network reorganization is related to emotion regulation processes. Mindfulness interventions have been particularly effective in promoting adaptive emotional regulation strategies [55], especially in affective disorders such as depression or anxiety [114]. Current findings indicate that mindfulness practice would facilitate the cognitive reappraisal of negative experiences and savoring of positive ones [115]. Consistent with the emphasis placed on emotional regulation process in mindfulness programs, we found that, while cognitive reappraisal was related to rumination, thought suppression and non-reactivity at pre-MBSR, it was related to mindfulness and well-being measures after the MBSR, which belongs to a more naturally adaptive cluster. Furthermore, community analysis detected that, after the MBSR, cognitive reappraisal was clustered with some mindfulness measures, such as decentering, interoceptive awareness, and observing. These results emphasize the importance of cognitive aspects of emotion regulation [116] as a key mechanism of MF interventions, which enriches our understanding of mindful-emotion regulation processes [117].

A final observation on the pre-post reorganization of constructs is related to well-being. Different psychological traditions emphasize the importance of consciousness in well-being promotion [118]. In this sense, mindfulness practice may promote well-being through a better awareness of basic psychological needs [119]. Current studies indicate that mindfulness training mediates positive well-being outcomes [120] and our NA supported these findings. Our results indicated that while well-being measures were quite disconnected from each other before the intervention, they increased their mutual relations after the MBSR, enhancing their relations with self-compassion and mindfulness nodes. Furthermore, network inference of centrality parameters showed that general well-being was one of the most central nodes in the network both before and after MBSR. Finally, community analysis detected a distinctive “Psychological well-being” cluster that included general well-being, life satisfaction and optimism both before and after MBSR. Summarizing, this NA seems to indicate that well-being, which is one of the main motivation of participants in meditation training [121], is one of the key component in MBSR and, according to other studies, it could be a factor that triggers cognitive changes in the process of MF trainings [122]. Thus, this psychonectome perspective provides new insights on the central of role psychological well-being in mindfulness.

The third hypothesis stated that, compared to the pre-MBSR state, mindfulness, emotion regulation and well-being constructs would increase their centrality in the resulting post-MBSR psychonectome. Consistent with previous theoretical models of mindfulness functioning [52], Expected Influence and Strength centrality indexes revealed that some measures of mindfulness (i.e. non-reactivity, interoceptive awareness, non-attachment, decentering and mindfulness self-compassion) were the most central in the networks both before and after the MBSR, together with a general well-being measure. Furthermore, emotional reappraisal and reflective cognitive style also emerged as central nodes in Expected Influence analysis. On the other hand, the nodes with the highest topological change after the MBSR were attentional control, compassion measures, depression and thought suppression. Centrality can be considered an indirect indicator of clinical significant changes as it is assumed that highly central constructs can influence other nodes in the network [123]. Although there is some concerns about which could be the best central indices in behavioral research [102,103], if the assumption is correct, the design of health promotion strategies and treatments should consider the convenience of prioritizing interventions on these central nodes [123,124]. Based on our centrality results, it could be hypothesized that MBSR interventions should focus on increasing trait-mindfulness levels and attentional control skills, together with compassion elements, and enhancing positive emotional regulation strategies, whereas they should reduce rumination tendencies. Enhancements in these elements seem to be topologically connected to well-being components. Although peripheral nodes are also relevant in the network, the centrality analyses suggest that intervening on the most central nodes might have stronger and faster dispersion to the whole system than intervening on peripheral ones [125]. Further empirical research should directly target these innovative variations in MF interventions.

At this point, it is important to take into account that stability analysis indicated that our MBSR network interpretations were reliable, as the results indicated that centrality indexes and the network interpretation would remain stable and accurate in other samples with fewer observations. Furthermore, predictability analysis also suggested that the overall predictability was quite high in both pre and post MBSR networks.

Finally, the fourth hypothesis of the current study was that MBSR would have an effect in regrouping the constructs into psychologically meaningful sub-networks. As a previous requirement for the interpretability of network changes, several analyses showed that there was true reorganization of the networks (see Supplementary materials), which provides robustness to the findings. Community analysis detected six node communities in pre-MBSR network and five node communities in post-MBSR network, thereby showing that the network was reorganized after the MBSR program. Specifically, whereas the pre-MBSR communities were composed by rather heterogeneous elements corresponding to different families of constructs (e.g. mindfulness and self-compassion constructs were scattered in different clusters in pre-MBSR network), the communities of constructs that emerged after the MBSR seemed to be reorganized in a more psychologically meaningful mode. Interestingly, this new reorganization corresponded more closely to the a priori five theoretical domains of constructs that were initially selected based on the available empirical evidence (see Table 2 and Fig 3).

The study has some strengths and limitations. Some of the strengths are that is a novel study in different ways. As far as we know, this is the first empirical study using NA to explore a standardized mindfulness intervention and we took this opportunity as a proof-of-concept of the ‘*psychonectome’*, using psychological constructs instead individual items (e.g. symptoms). It also included, in the same design, a variety of a comprehensive list of constructs that the literature has found to be relevant for the practice of meditation. Also, the analyses conducted in the study have included some of the most recent procedures proposed to analyze and visualize networks in Psychology (e.g., Expected Influence as a measure of centrality, which seems more adequate than others, like strength, or Predictability, to analyze the explained variance of each node in the network) and incorporate some additional indexes and analyses (e.g., Efficiency and clustering), that are typically used to analyze brain activity [36].

Yet, these initial results must be considered in the light of some methodological limitations. First, our sample consisted of general population individuals voluntarily attending a MBSR course. As an important aspect of meditation practice is to be motivated to engage in its practice [52,69], the lack of random allocation to the treatments is an acknowledged limitation in this field of research [126–128]. A second limitation was that we included both, participants with and without previous meditation experience from different traditions, which might be a relevant moderator of our results [44]. Given the novelty of our study, our goal was to examine how a mindfulness intervention, in general, would impact on the relations between different psychological constructs from a psychonectome perspective. Future studies should compare the network reorganization between different meditation practices (e.g. mindfulness vs. compassion meditation), between different meditation experience (e.g. novices vs. experts) as well as adding a control group. A third limitation is that only self-reported measures were included in the networks. Future studies should include other type of elements like behaviors [129], biological parameters [125], performance in cognitive experimental tasks [130], or even external factors to the network as, for instance, life stressors [14]. Including further relevant information in networks might shed light on the action mechanisms underlying the practice of meditation and, in general, any intervention. A fourth limitation is that only pre and post information was included. Future studies on networks analysis within this field should consider adding longitudinal data from inter-session measures and follow-ups, as it has already been done in MBSR interventions [122], to infer causality in the psychonectome [131,132]. Finally, it should be also taken into account that NA methods are still relatively new in psychology and there is still no consensus on issues as relevant as the best procedure to estimate the required sample size to obtain accurate edge weights [124], the optimal analytic procedures [93,133], or the most accurate and reliable indexes of centrality [94,102,134]. More specifically, there is a current debate on the replicability of findings in NA [133,135,136]. Although replicability can be partially improved by implementing good practices like providing R scripts and data matrices, which has been done in the present study, this practice has been barely done in most of the published literature in psychopathology, as a recent meta-analysis has found [13]. Also, and more importantly to increase replicability, it could be possible that using only signs and symptoms in network analysis, instead of psychological constructs, may limit the statistical accuracy of the networks [22]. Yet, it is still soon to provide definite answers on the incremental validity of NA as compared to more traditional ways of analyzing the relationships between psychological attributes [21,22].

Despite these limitations, this study provides some novel results on the complex multivariate interaction of the variables involved in mindfulness practice which is revealed by network analysis. Beyond these specific results, the current ‘psychonectome’ proof-of-concept approach seems useful to provide further evidence of the mind as a complex network of psychological constructs. The approach of considering topological aspects of the relations between constructs may help to enhance our understanding of psychological functioning as a complex network of interacting elements that are mutually interconnected [18–20,137,138]. This unique perspective has become central in fields like neuroscience [25,35] and is likely that can also provide useful insights on the functioning and mechanisms of human mind.

## Supporting information

S1 Fig Principal components analysis network configurations of Pre- (panel A) and Post-MBSR intervention (panel B).
(TIF)

S2 Fig Pre- (panel A) and Post-MBSR intervention (panel B) predictability.
(TIF)

S3 Fig Pre- and Post-MBSR intervention predictability scores (i.e., percentage of variance for each node of the network). (TIF)

S4 Fig 95% bootstrapped CIs around each edge-weight for the estimated networks of pre-MBSR (left) and post- MBSR (right). (TIF)

S5 Fig Pre-MBSR (Panel A) and post- MBSR (Panel B) stability of centrality indices, showing an average correlation between the centrality indices of the original sample with people dropped.
(TIF)
