# Supplementary figures and images for: Does mindfulness change the mind? A novel psychonectome perspective based on Network Analysis
Source: PLoS One. 2019 Jul 18;14(7):e0219793. doi: 10.1371/journal.pone.0219793 (PMC6638953; doi:10.1371/journal.pone.0219793)

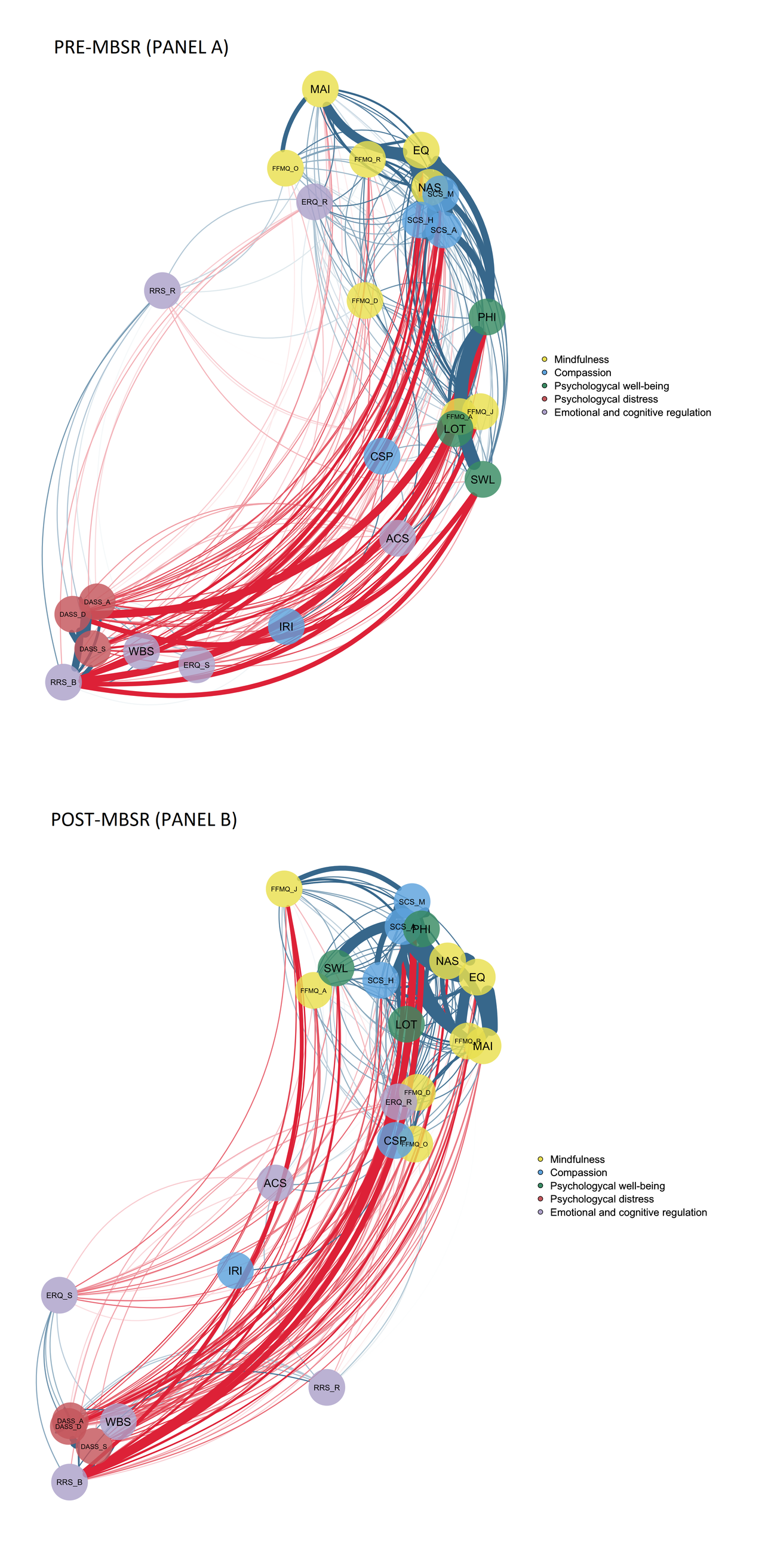

Supplement: S1 Fig — Principal components analysis network configurations of Pre- (panel A) and Post-MBSR intervention (panel B). (TIF) [file pone.0219793.s001.tif]

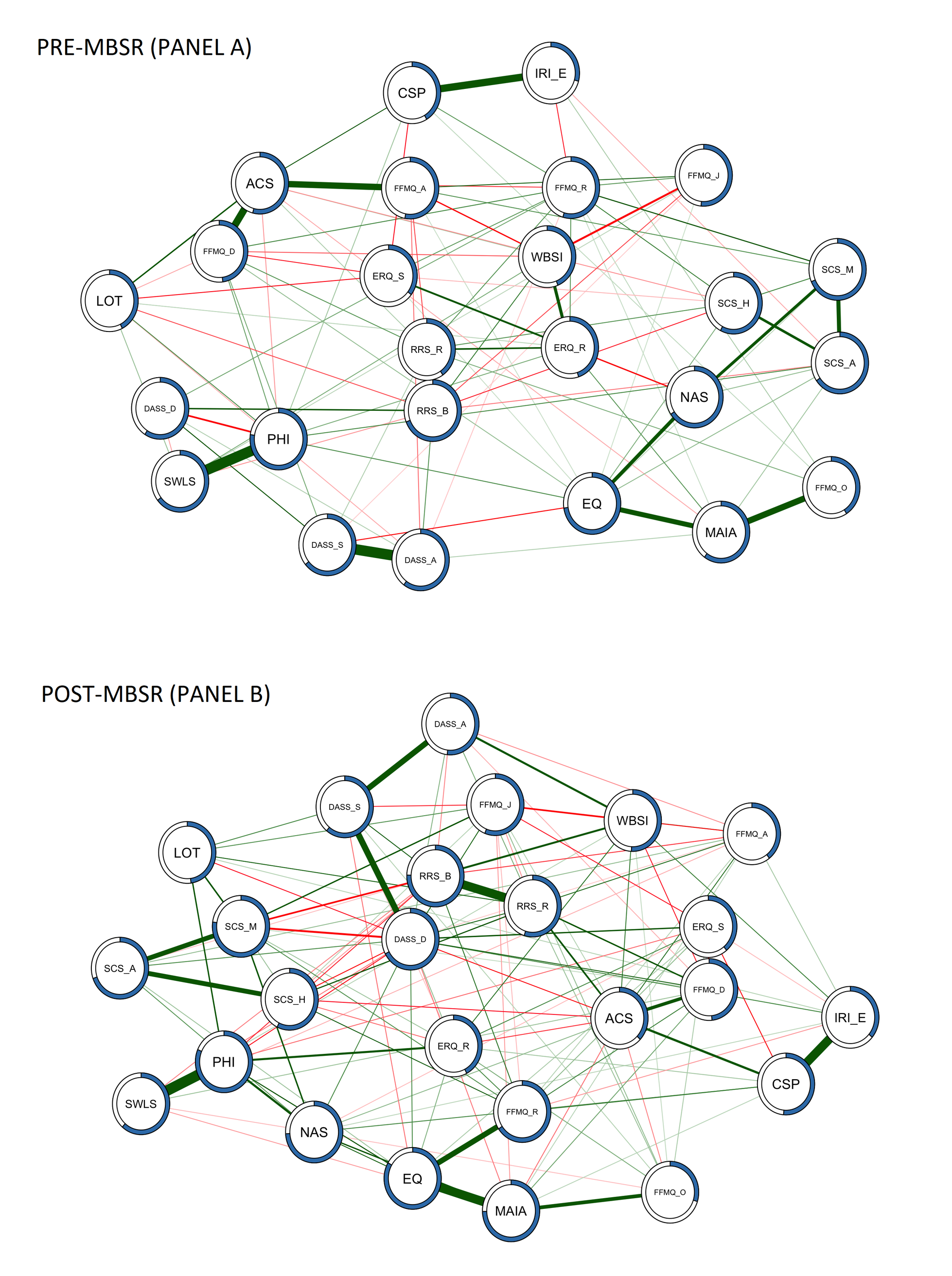

Supplement: S2 Fig — Pre- (panel A) and Post-MBSR intervention (panel B) predictability. (TIF) [file pone.0219793.s002.tif]

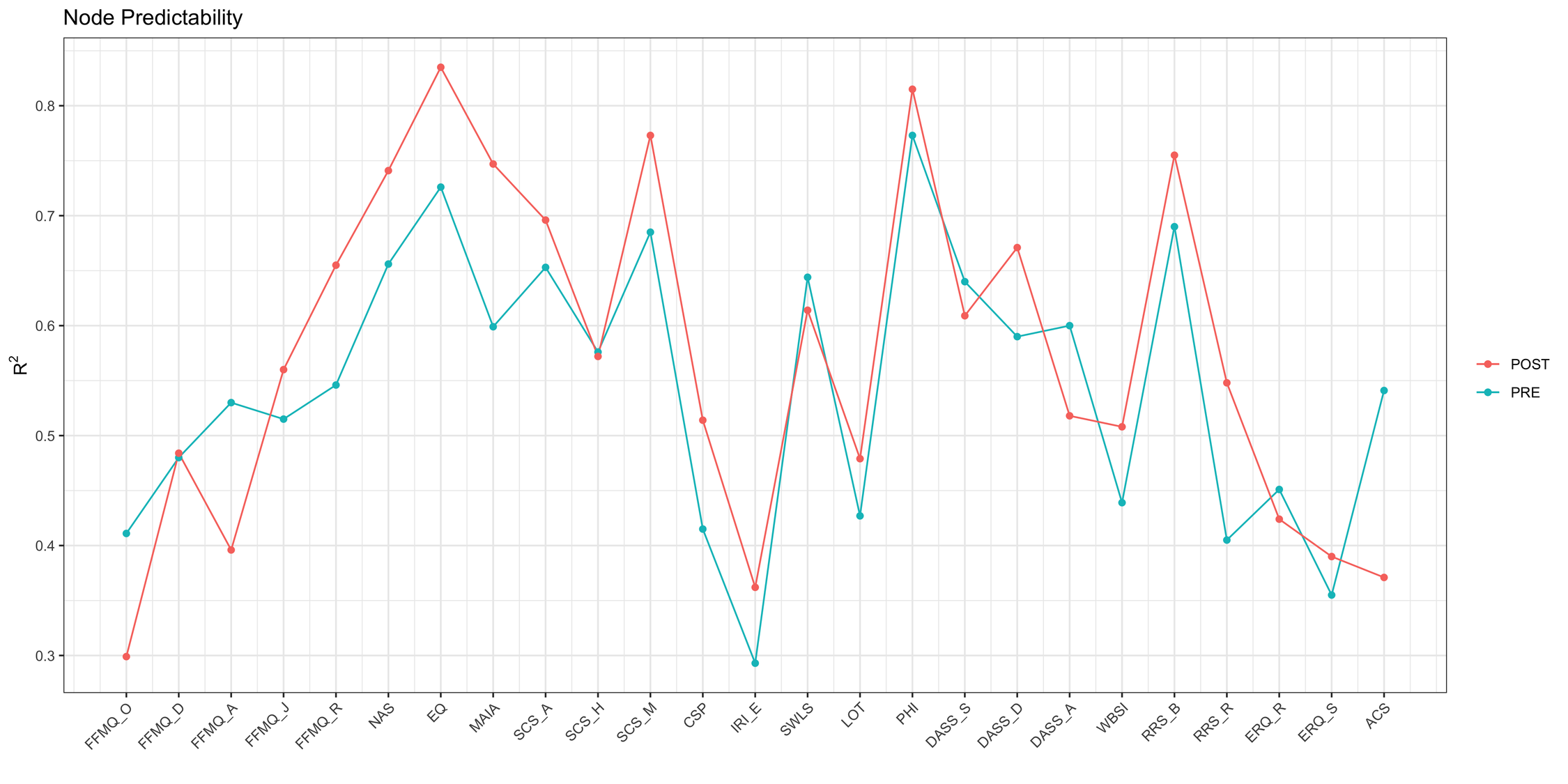

Supplement: S3 Fig — (TIF) [file pone.0219793.s003.tif]

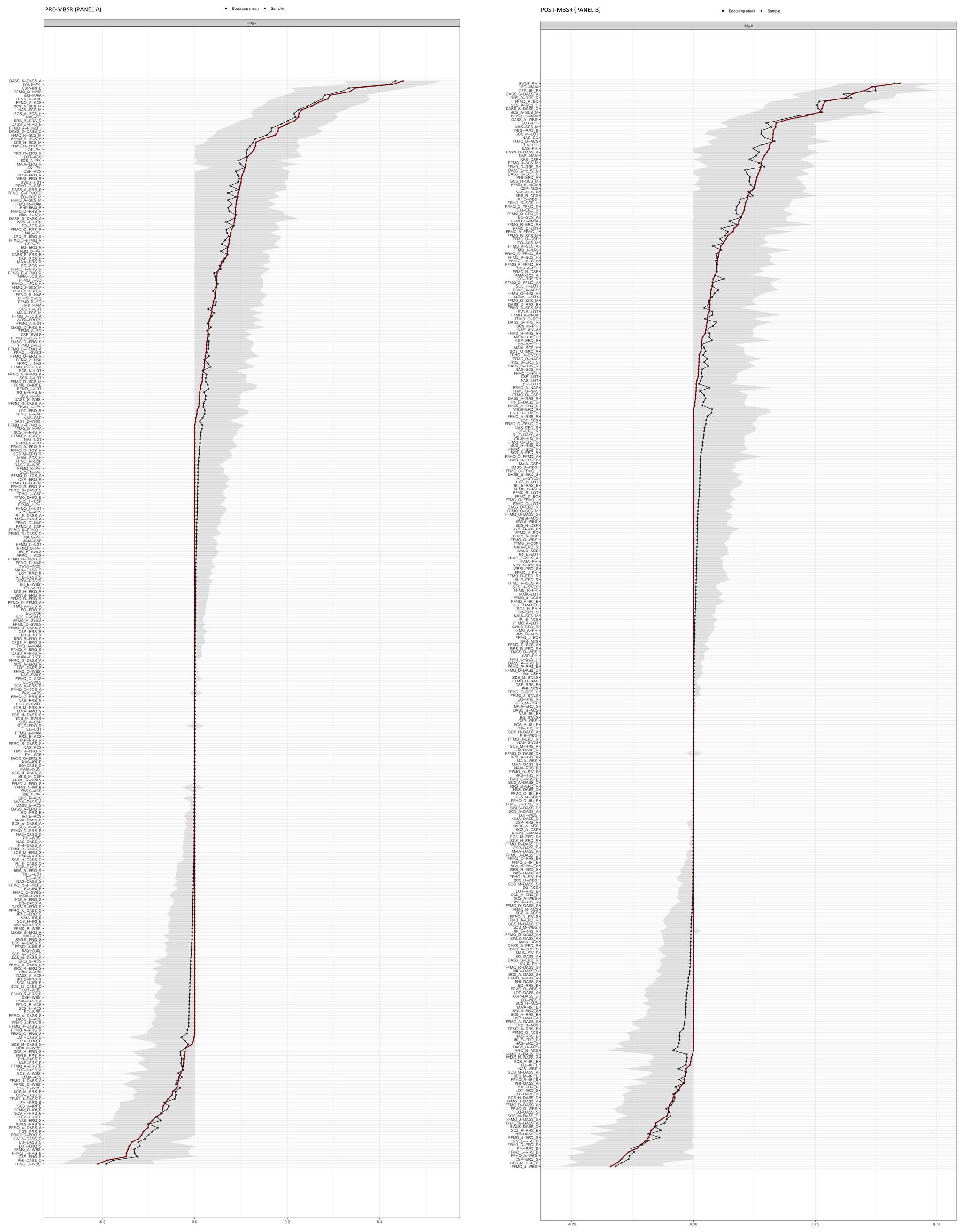

Supplement: S4 Fig — (TIF) [file pone.0219793.s004.tif]

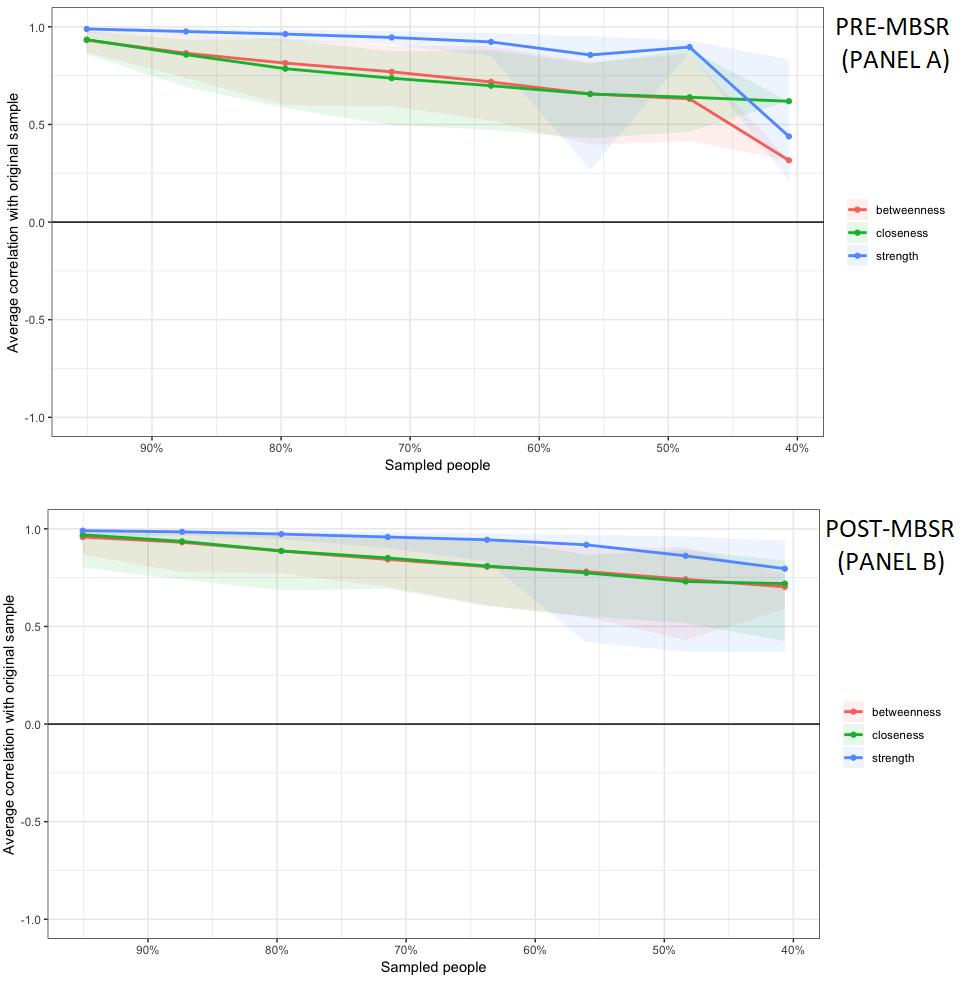

Supplement: S5 Fig — Pre-MBSR (Panel A) and post- MBSR (Panel B) stability of centrality indices, showing an average correlation between the centrality indices of the original sample with people dropped. (TIF) [file pone.0219793.s005.tif]
